# 15-Deoxy-Delta-12,14-Prostaglandin J_2_ Inhibits Lung Inflammation and Remodeling in Distinct Murine Models of Asthma

**DOI:** 10.3389/fimmu.2017.00740

**Published:** 2017-06-30

**Authors:** Diego S. Coutinho, Edna A. Anjos-Valotta, Caio V. M. F. do Nascimento, Ana Lucia A. Pires, Marcelo H. Napimoga, Vinícius F. Carvalho, Rafael C. Torres, Patrícia M. R. e Silva, Marco A. Martins

**Affiliations:** ^1^Laboratório de Inflamação, Instituto Oswaldo Cruz, Fundação Oswaldo Cruz, Rio de Janeiro, Brazil; ^2^Universidade Estadual da Zona Oeste, Rio de Janeiro, Brazil; ^3^Laboratory of Immunology and Molecular Biology, São Leopoldo Mandic Institute and Research Center, Campinas, Brazil

**Keywords:** asthma, house dust mite, allergen, 15d-PGJ_2_, lung inflammation, resolution

## Abstract

15-deoxy-Δ-12,14-prostaglandin J_2_ (15d-PGJ_2_) has been described as an anti-inflammatory lipid mediator in several *in vitro* and *in vivo* studies, but its effect on allergic pulmonary inflammation remains elusive. The aim of this study was to investigate the therapeutic potential of 15d-PGJ_2_ based on distinct murine models of allergic asthma triggered by either ovalbumin (OVA) or house dust mite extract (HDM). Characteristics of lung inflammation, airway hyper-reactivity (AHR), mucus exacerbation, and lung remodeling in sensitized A/J mice treated or not with 15d-PGJ_2_ were assessed. 15d-PGJ_2_ treatments were carried out systemically or topically given *via* subcutaneous injection or intranasal instillation, respectively. Analyses were carried out 24 h after the last allergen provocation. Irrespective of the route of administration, 15d-PGJ_2_ significantly inhibited the peribronchial accumulation of eosinophils and neutrophils, subepithelial fibrosis and also mucus exacerbation caused by either OVA or HDM challenge. The protective effect of 15d-PGJ_2_ occurred in parallel with inhibition of allergen-induced AHR and lung tissue production of pro-inflammatory cytokines, such as interleukin (IL)-5, IL-13, IL-17, and TNF-α. Finally, 15d-PGJ_2_ was found effective in inhibiting NF-κB phosphorylation upon HDM challenge as measured by Western blotting. In conclusion, our findings suggest that 15d-PGJ_2_ can reduce crucial features of asthma, including AHR, lung inflammation, and remodeling in distinct murine models of the disease. These effects are associated with a decrease in lung tissue generation of pro-inflammatory cytokines by a mechanism related to downregulation of NF-κB phosphorylation.

## Introduction

Atopic asthma is a chronic inflammatory disease of the lung airways, triggered by a combination of genetic predisposition and environmental allergens, such as pollen, air pollution, and the fecal matter from dust mites and cockroaches (1–3). The prevalence of asthma has increased over the last 50 years, affecting 5–20% of the population worldwide and causing 250,000 annual deaths globally (3, 4). Asthma pathogenesis is driven by T cells and T-helper 2 (Th2) cytokines, resulting in eosinophil infiltration into lung tissue, peribronchiolar fibrosis, thickening of airway wall layers, epithelial goblet cell metaplasia, and AHR (5, 6). Asthma affects children and adults of both sexes cutting down on the patient’s ability to breathe. Currently, the asthma therapy is based on inhaled anti-inflammatory steroids and β2 adrenergic agonists, which control the symptoms of the disease quite effectively (7–9). However, some asthmatics are insensitive to glucocorticoids and present adverse effects, indicating the need to develop new therapy alternatives safe and effective in controlling the disease (10–12).

Peroxisome proliferator-activated receptor gamma (PPAR-γ) is an intracellular receptor initially characterized as a regulator of adipocyte differentiation. The expression of PPAR-γ is apparent in numerous inflammatory cells, including macrophages, T cells, and eosinophils. PPAR-γ acts as a transcription factor and its activation inhibits the development and perpetuation of the inflammatory response (13–16). The endogenous ligand 15-deoxy-Δ^-12,14-^prostaglandin J_2_ (15d-PGJ_2_) and synthetic thiazolidinedione derivatives (TZDs) activate PPAR-γ (17). Prior studies revealed that TZDs inhibit AHR and lung inflammation in murine models of asthma (13, 17, 18). Nevertheless, these drugs present a range of adverse effects, including hepatotoxicity, weight gain, heart failure, pseudo-anemia, myalgias, and bone fractures, leading to discontinuation of several drugs of this class in phase II clinical trials (19, 20).

Since TZDs present a vast spectrum of adverse effects, an alternative might be an endogenous activator of the PPAR-γ receptor, such as 15d-PGJ_2_ (21, 22). In fact, 15d-PGJ_2_ acts on the resolution of inflammation protecting cells and tissues in inflamed sites, through mechanisms such as blockade of leukocyte infiltration (23) and apoptosis of infiltrated polymorphonuclear cells (24). Furthermore, there is a close relationship between decrease of inflammatory markers and presence of endogenous 15d-PGJ_2_ in murine models of intestinal ischemia and reperfusion (25) and zymosan-induced peritonitis (26). Nevertheless, differently from the scenario of many studies on the anti-asthma effects of synthetic PPAR-γ ligants, nothing is known so far on the effectiveness of 15d-PGJ_2_ in *in vivo* settings of pulmonary asthmatic changes. Thus, the aim of this study was to evaluate the consequence of interventional therapeutic treatments by 15d-PGJ_2_ on pivotal asthma changes, such as inflammatory infiltrate, airway hyperreactivity, mucus exacerbation, and lung remodeling, following exposure of mice to distinct allergens. Since NF-κB pathway is highly expressed in severe asthmatics (27) and is crucial for the generation of pro-inflammatory cytokines, such as interleukin (IL)-13 and TNF-α (28, 29), this study also evaluated the putative implication of NF-κB in the mechanism of action of 15d-PGJ_2_.

## Materials and Methods

### Animals

Male mice of strain A/J (18–20 g) were obtained from the Oswaldo Cruz Foundation (Rio de Janeiro, Brazil) breeding unit and kept in the animal care facility of Oswaldo Cruz Institute. The animals were maintained in ventilated cages (five mice per cage) under pathogen-free conditions in a room at 20–24°C, relative humidity (40–70%) on a 12 h light/dark cycle with water, and food *ad libitum*. The Animal Ethics Committee of the Oswaldo Cruz Foundation approved all procedures involving care and use of animals in this study (license no. LW 23/10).

### Ovalbumin (OVA) Asthma Model and Treatment Protocol

**Figure 1:**
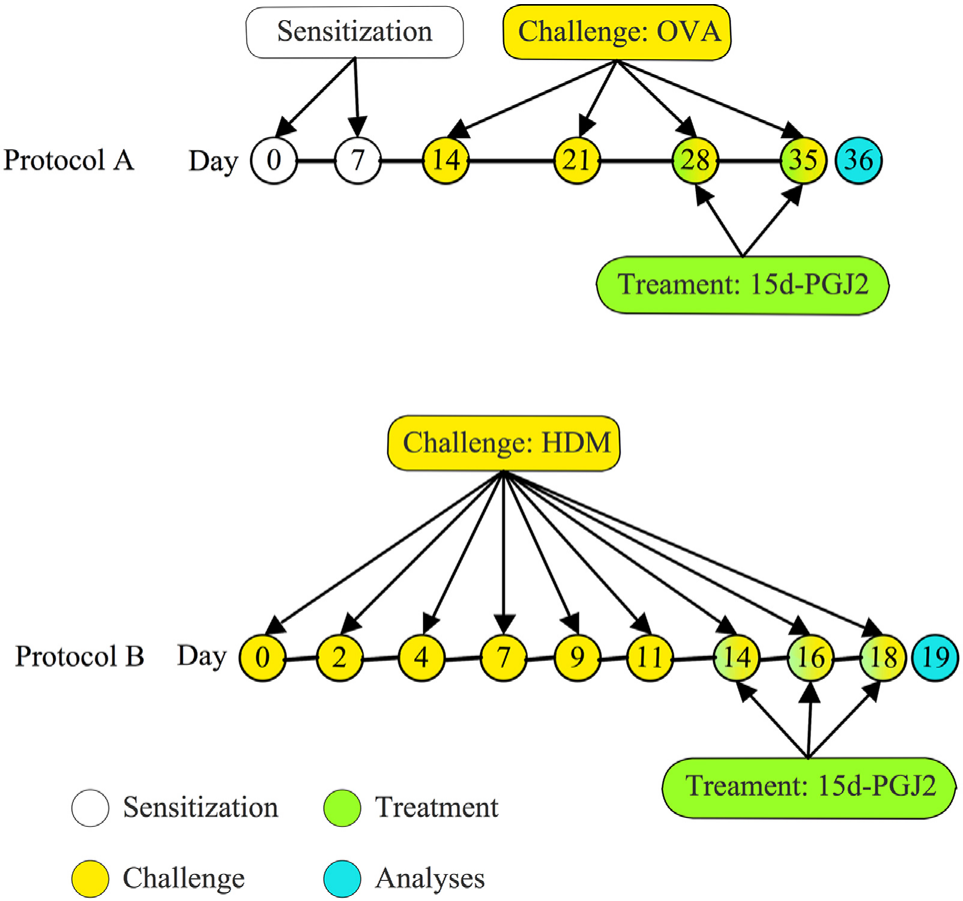
Treatment protocols. **(A)** Mice were sensitized at days 0 and 7 and subjected to a series of four provocations with ovalbumin (OVA) (50 μg/25 μL) at days 14, 21, 28, and 35 post-sensitization. 15d-PGJ_2_ (30 or 100 μg/kg) was subcutaneously injected at days 28 and 35, 30 min before OVA; **(B)** mice were challenged with house dust mite extract (HDM) (15 μg/15 μL/mouse) intranasally, three times per week during 3 weeks. Treatments with 15d-PGJ_2_ occurred at the last week, as administered subcutaneously (30–100 μg/kg) or intranasally (0.7–2.3 μg/25 μL/mouse), 30 min before provocation.

A/J mice were actively sensitized subcutaneously on day 0 and boosted 7 days later with 50 µg of OVA adsorbed to 5 mg of Al(OH)_3_ in 200 µl of sterile 0.9% saline. One week after receiving booster, the animals were anesthetized with isoflurane and given OVA (50 μg/25 μl of saline), or vehicle, *via* an intranasal instillation once a week for 4 weeks (5, 30). 15d-PGJ_2_ interventional treatment was done subcutaneously (30 or 100 μg/kg) 30 min before OVA provocation only at the third and fourth provocation. All analyses were done 24 h after last provocation (Figure 1A).

### House Dust Mite Extract (HDM) Asthma Model and Treatment Protocol

A/J mice were anesthetized with isoflurane and given 15 µl HDM (15 µg of *Dermatophagoides pteronyssinus* extract) or phosphate-buffered saline (PBS), 3 days a week for 3 weeks, *via* intranasal instillation (Figure 1B). 15d-PGJ_2_ was given subcutaneously (30–100 μg/kg) or intranasally (0.7–2.3 μg/mouse), 30 min before HDM, at the third week of allergen exposure. All analyses were made 24 h after the last allergen challenge. Since there is substantial evidence for the existence of a genetic asthma-prone background for A/J mice (31, 32), these animals were selected for sensitization and challenge in both ovalbumin and HDM systems.

### Measurement of Airway Hyperreactivity

For transpulmonar resistance and elastance assessments, mice were anesthetized (nembutal 60 mg/kg, intraperitoneal), tracheostomized, and subjected to neuromuscular blockade (Rocuronium bromide, 1 mg/kg, intravascular), before being mechanically ventilated in a Buxco FinePoint R/C system (Buxco Electronics, Sharon, CT, USA). Changes in lung resistance (cm H_2_O ml^−1^ s^−1^) and elastance (cm H_2_O ml^−1^) in each breath cycle were calculated based on airflow and pressure signals. Measurements were taken at baseline, after aerosolized PBS, and after increased concentrations of aerolized methacholine (3, 9, and 27 mg/ml) as reported (5, 33).

### Histological Analysis

After lung function evaluation, mice were killed under terminal anesthesia (Nembutal 200 mg/kg, intravenous), and the lung lobes were removed, and, immediately, fixed in formalin-Milloning for preservation of pulmonary architecture and posterior inclusion in paraffin. Sections of lung tissue (4 µm) were stained with sirius red (Direct red 80, CI 35780; Aldrich, Milwaukee, WI, USA) pH 10.2 for quantification of leukocyte infiltration. In an alkaline pH, Sirius red is able to selectively stain eosinophil cytoplasm in red, contrasting with a pale cytoplasmic background of mononuclear cells and neutrophil granulocytes, which could be characterized according to their typical morphology (34, 35). Mononuclear cells, eosinophils, and neutrophils were quantified around the bronchiolar airway with the help of a morphometric reticle (with a 10^4^ µm^2^ area) attached to the eyepiece of a light microscope. We randomly took 7–10 airways in the peribronchiolar region to count the target cells. Photomicrographs of representative distal airways were obtained in the 1000× magnification under light microscopy.

Histologic sections (4 µm) were stained with hematoxylin and eosin stain and periodic acid-Schiff stain (Periodic Acid-Schiff Staining System, Sigma-Aldrich) for measuring mucus production (36). Hypertrophy and hyperplasia of goblet cells were assessed by the analysis of area occupied by goblet cells in bronchioles. Both mucus production and goblet cell hypertrophy/hyperplasia were analyzed in distal airway photomicrographs (400× *magnification)* (7–10 distal airways per lung) under light microscopy.

Peribronchiolar fibrotic response was evaluated in lung tissue sections (4 µm) stained with Gömöri trichrome and counterstained with hematoxilin and eosin. Trichrome stains fibrous components of extracellular matrix in a characteristic blue–green color (5, 37). The area of peribronchiolar trichrome staining was outlined and quantified for the total deposition of the extracellular matrix in that microenvironment. The evaluation was made in an image analyzer system (Image-Pro^®^ Plus, 4.1; Media Cybernetics, Houston, TX, USA) using photomicrographs (7–10 distal airways per lung) obtained from a light microscope at a magnification of 200×.

### Cytokine and Chemokine Measurements

After lung function evaluation, mice were killed under terminal anesthesia (as reported above), and the left lung lobes were removed, immediately frozen in liquid nitrogen, and stored at −80°C. Commercial enzyme immunosorbent assay (ELISA) kits were used for the measurement of cytokine and chemokine proteins in whole-lung homogenates and cell-free supernatants as reported (38). Briefly, lung tissue was homogenized on ice using a tissue homogeneizer (Omni International, Kennesaw, GA, USA) in 1 ml PBS containing 0.05% Triton X-100 and a protease inhibitor cocktail (Hoffmann-La Roche, Basel, Switzerland). The resulting supernatants were isolated after centrifugation (10,000 × *g*, 15 min, 4°C). Samples were quantified using commercially available kits [IL-5, IL-13, IL-17 and TNF-α; Duoset, R&D Systems, MN, USA] and eotaxin-1 (R&D Systems), according to the manufacturer’s instructions.

### Western Blotting Analysis

Pulmonary tissue was homogenized in 500 µl of an ice-cold lysis buffer containing a cocktail of protease inhibitors, in addition to 0.1% Triton X-100 in PBS 1×. The homogenates were centrifuged at 13,000 × *g* for 10 min at 4°C and the supernatant was collected for quantification of total proteins and expression of transcription factors. Samples with equal protein concentration (100 μg/lane) were separated on a 10% SDS-PAGE gel and transferred to PVDF membranes (GE Healthcare, Little Chalfont, UK). Membranes were incubated to either NF-κB p65 and β-actin specific mouse monoclonal antibodies or pNF-κB p65 specific rabbit polyclonal antibodies (1:1,000 dilution; Santa Cruz Biotechnology, CA, USA), followed by incubation with a HRP-conjugated secondary antibody (1:10,000 dilution; Santa Cruz Biotechnology, CA, USA) for 1 h at room temperature. The protein expression was detected using enhanced chemiluminescence. The band intensity was quantified by densitometry using Image J Analysis program (Research Services Branch).

### RBL-2H3 Cell Culture, Stimulation, and Treatments

Wistar rat basophilic cell lineage (RBL-2H3) was kept at 37°C in a humidified 5% CO_2_: 95% air atmosphere in Dulbecco’s modified Eagle’s medium (DMEM), supplemented with 15% fetal bovine serum, penicillin 100 U/ml, and streptomycin 0.1 mg/ml as reported (39). The cells were sensitized with IgE anti-DNP antibody (1 µg/ml) at 37°C for 20 h in a humidified 5% CO_2_: 95% air atmosphere. Then, the medium was replaced by Tyrode assay buffer (119 mM NaCl, 4.7 mM KCl, 2.5 mM CaCl_2_, 1.2 mM MgSO_4_, 10 mM 4-2-hydroxyethyl-1-piperazineethane sulfonic acid, 5 mM glucose, and 0.1% (w/v) BSA, pH 7.3), and the cells were treated with 15d-PGJ_2_ alone or in combination with the PPAR-γ antagonist GW9662 for 1 h. After treatment, RBL-2H2 cells were stimulated with antigen (DNP-BSA, 10 ng/ml) for 45 min at 37°C in 5% CO_2_: 95% air atmosphere. After centrifugation at 150 *g* for 10 min, the supernatant was collected and β-hexosaminidase (β-hex) release was quantified as reported (40). This method consists of a reaction of β-hex in sample with 1 mM p-nitrophenyl *N*-acetyl-β-d-glucosamide in 0.1 M sodium citrate buffer (pH 4.5) for 50 min at 37°C. Then, the reaction was stopped using 0.2 M glycine and reaction measured using a SpectraMax M5 (Molecular Devices, Sunnyvale, CA, USA) spectrophotometer at 405 nm.

### Drugs and Reagents

*Dermatophagoides pteronyssinus* extract was purchased from GREER Laboratories (NC, USA). 15d-PGJ_2_ and GW9662 were purchased from Cayman Chemical Company (MI, USA). OVA (Grade-V), methacholine, and nembutal were purchased from Sigma-Aldrich (MO, USA) and Rocuronium bromide and isoflurane from Cristália (Rio de Janeiro, Brazil). All drugs were freshly prepared before use.

### Statistical Analysis

The statistical analysis was performed using the Prism package Graph-Pad Software (version 5.0, San Diego, CA, USA). Data were expressed as mean ± SEM. Tests were carried out using one-way ANOVA followed by the Newman–Keuls–Student test, or two-way ANOVA followed by the Bonferroni test. *P* ≤ 0.05 was considered significant.

## Results

### Effects of 15d-PGJ_2_ on OVA-Induced AHR, Lung Inflammation, and Remodeling in Mice

**Figure 2:**
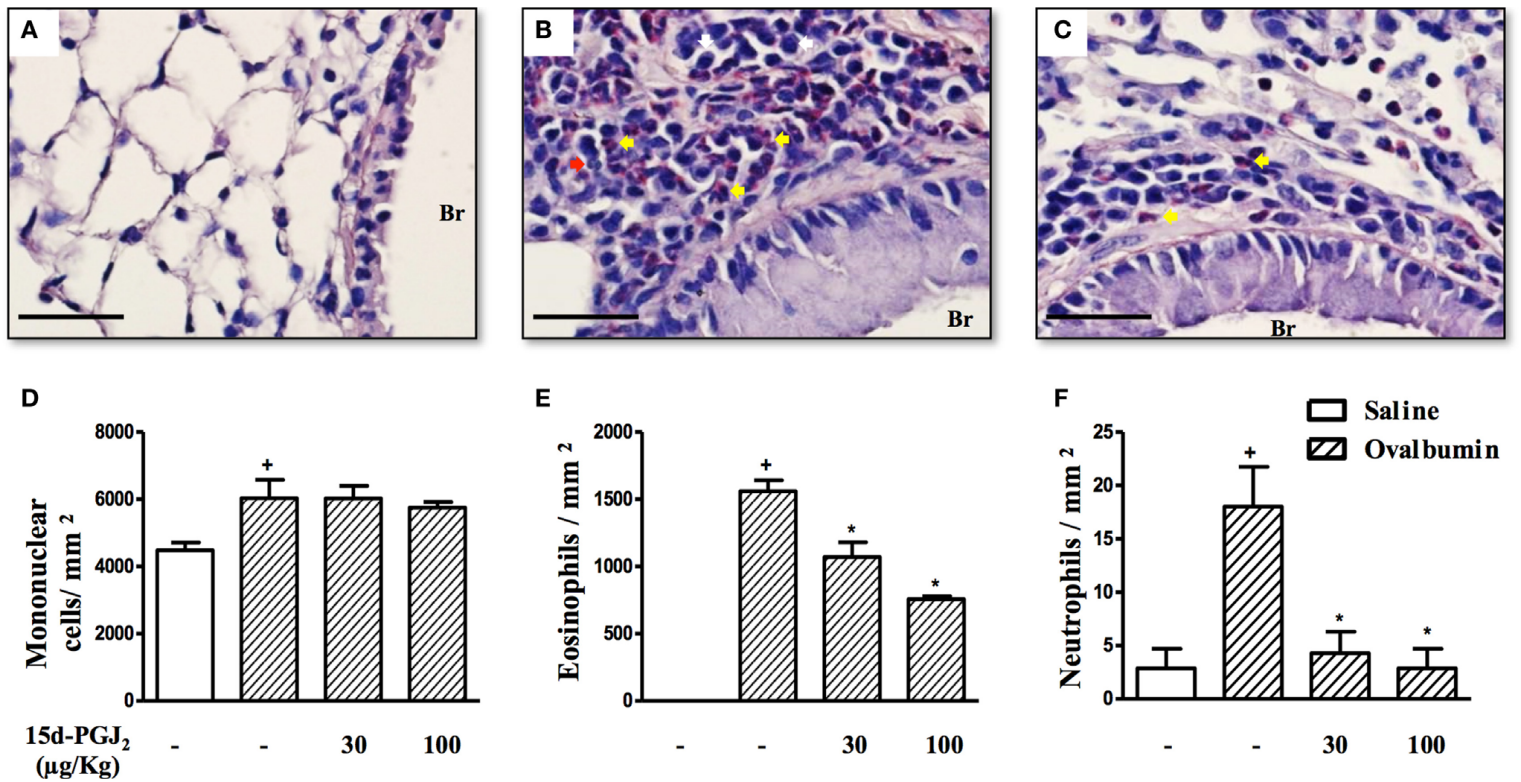
15d-PGJ_2_ reduces peribronchiolar infiltration of eosinophils and neutrophils induced by ovalbumin (OVA) challenge. Photomicrographs of representative airways in Sirius Red-stained lung sections from mice challenged with saline **(A)**, OVA **(B)**, and OVA + 15d-PGJ_2_ (100 μg/kg, s.c.) **(C)**. White, yellow, and red arrows indicate mononuclear cell, eosinophil, and neutrophil, respectively. The number of mononuclear cells **(D)**, eosinophils **(E)**, and neutrophils **(F)** in peribronchiolar regions were counted in 7–10 bronchioles per mouse. Treatments were carried out 30 min before OVA exposure during the last 2 weeks of provocation. All samples for histologic examinations were undertaken 24 h after the last OVA challenge. Each value represents the mean ± SEM from seven animals. Br, bronchiolar lumen. Scale bar = 40 µm. ^+^*P* < 0.05 as compared to saline-challenged group; *P < 0.05 as compared to OVA-challenged group.

Ovalbumin provocation of actively sensitized mice, as indicated in Figure 1A, yielded a significant increase in the numbers of mononuclear cells, eosinophils, and neutrophils in peribronchiolar areas 24 h after the last challenge as compared to sham-challenged mice. Representative histological lung sections are shown in Figures 2A,B, whereas quantitative data are indicated in Figures 2D–F. As indicated in Figures 2C,D–F, these changes were clearly sensitive to the interventional treatment with 15d-PGJ_2_ (30 or 100 μg/kg, s.c.) carried out at the third and fourth week of OVA provocations as illustrated in Figure 1A.

**Figure 3:**
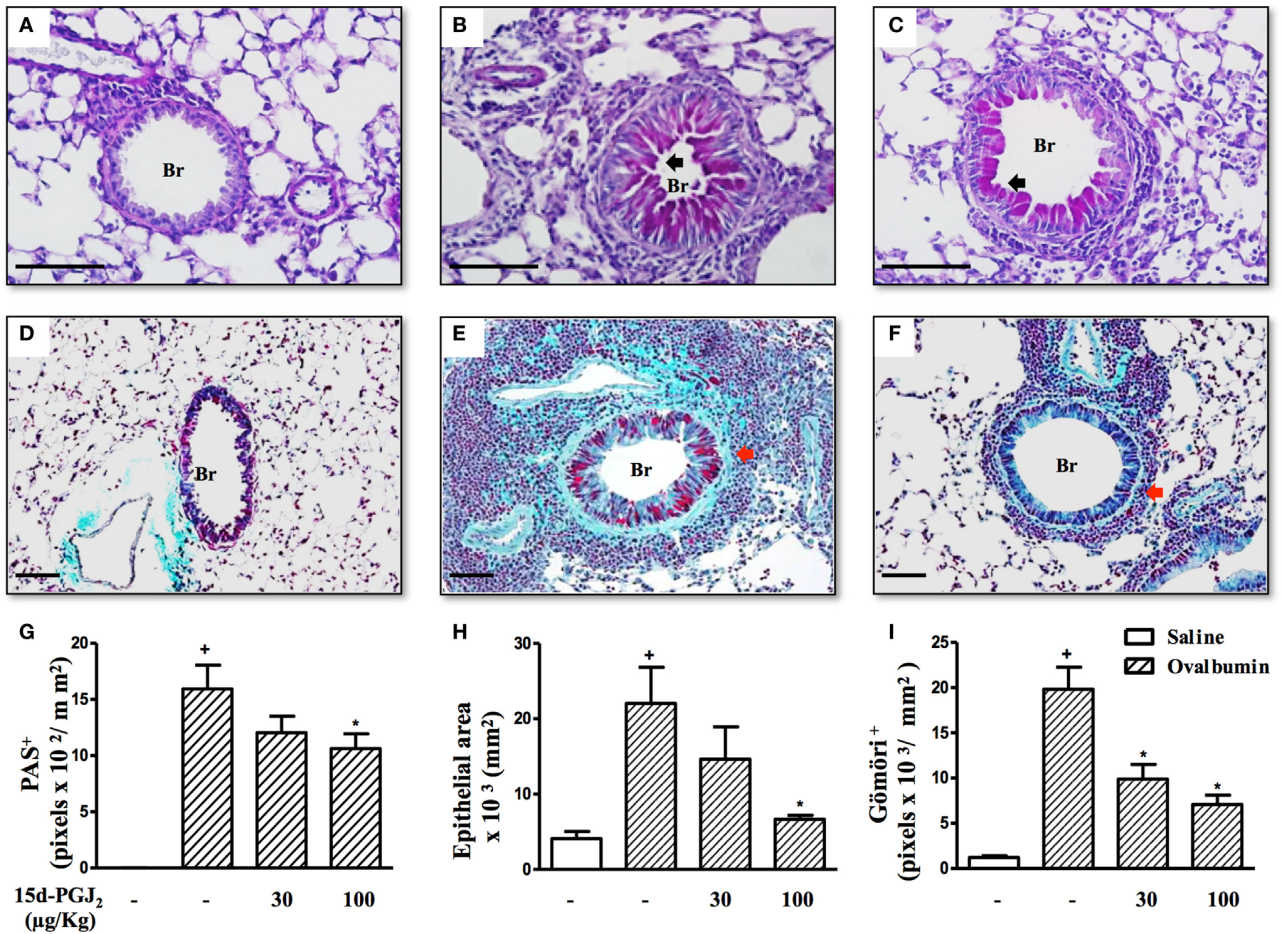
15d-PGJ_2_ decreases bronchiolar epithelium thickening, mucus production, and bronchiolar subepithelial fibrosis induced by ovalbumin (OVA) challenge. Photomicrographs of representative airways in PAS- or Gömöri Trichrome-stained lung sections from mice challenged with saline **(A,D)**, OVA **(B,E)**, and OVA + 15d-PGJ_2_ (100 μg/kg, s.c.) **(C,F)**. Black and red arrows indicate PAS and Gömöri Trichrome staining, respectively. Mucus production **(G)**, bronchiolar epithelium thickness **(H)**, and peribronchiolar fibrosis **(I)** were carried out by quantitative digital analysis in 7–10 bronchioles per mouse. Treatments were carried out 30 min before OVA exposure during the last 2 weeks of provocation. All samples for histologic examinations were undertaken 24 h after the last OVA challenge. Each value represents the mean ± SEM from seven animals. Br, bronchiolar lumen. Scale bar = 100 µm. ^+^*P* < 0.05 as compared to saline-challenged group; *P < 0.05 as compared to OVA-challenged group.

To access the putative effect of subcutaneous treatment of 15d-PGJ_2_ on lung remodeling triggered by OVA provocation, we have assessed distinct lung sections stained with PAS and Gömöri trichrome. Significant changes in mucus production, goblet-cell hypertrophy/hyperplasia, and peribronchiolar fibrosis were clearly apparent in OVA challenged mice as compared to sham-challenged ones. Representative photomicrographs are shown in Figures 3A,D (negative controls) and Figures 3B,E (positive controls), while quantitative data are shown in Figures 3G–I. The interventional treatment of 15d-PGJ_2_ (100 μg/kg, s.c.) significantly inhibited all these changes (Figures 3C,F,G–I, concerning quantitative data). OVA-induced peribronchiolar fibrosis was also inhibited by 30 µg/kg 15d-PGJ_2_.

**Figure 4:**
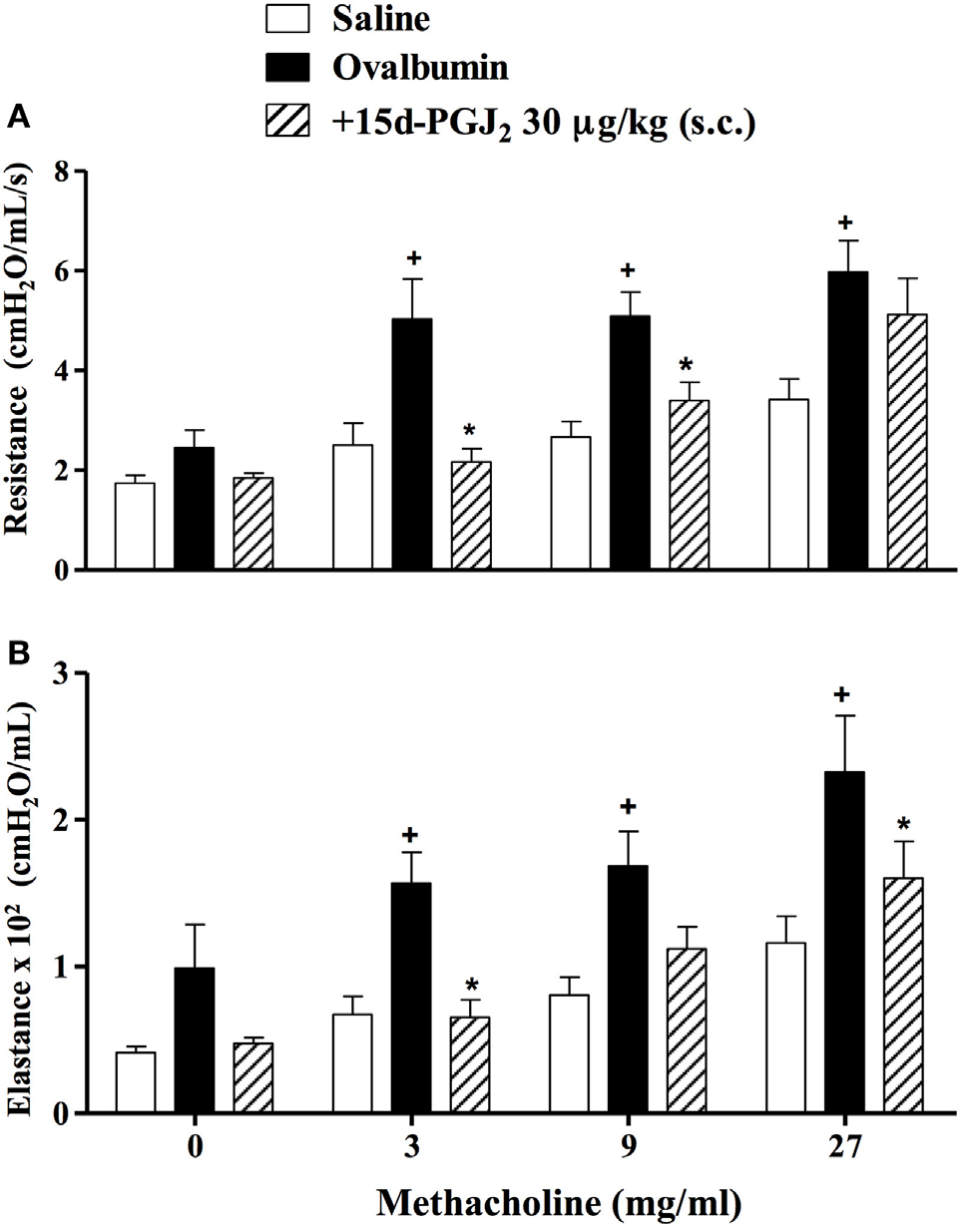
Subcutaneous administration of 15d-PGJ_2_ reduces ovalbumin (OVA)-induced bronchial hyperreactivity. Airway responses were measured as changes in lung resistance **(A)** and elastance **(B)** induced by increasing concentrations of methacholine (3–27 mg/ml). Treatment was carried out 30 min before OVA nasal instillation during the two last weeks of provocation. The analyses were undertaken 24 h after the last ovalbumin challenge. Each value represents the mean ± SEM from six to seven animals. ^+^*P* < 0.05 as compared to saline-challenged group; *P < 0.05 as compared to ovalbumin-challenged group.

To analyze the effect of 15d-PGJ_2_ on OVA-induced changes in lung functions, we used the invasive whole body barometric plethysmography. Exposure to OVA resulted in AHR, which was revealed by increased levels of airway resistance and lung elastance following aerolized methacholine (3–27 mg/ml), as compared to saline-challenged mice. 15d-PGJ_2_ at 30 μg/kg, s.c. decreased both airway resistance (Figure 4A) and elastance (Figure 4B).

As shown in Table 1, 15d-PGJ_2_ (100 μg/kg, s.c.) significantly inhibited the increased levels of IL-5, IL-13, and TNF-α in lung tissue homogenates of OVA-challenged mice. IL-5 levels were also sensitive to the dose of 30 μg/kg of 15d-PGJ_2_ (Table 1).

**Table 1:** Effect of 15d-PGJ_2_ on ovalbumin (OVA)-induced cytokine generation in the lung tissue of sensitized mice.

Cytokine (ρg/lung)	Saline	OVA	+15d-PGJ_2_ (30 µg/kg)	+15d-PGJ_2_ (100 µg/kg)
Interleukin (IL)-5	687 ± 83	1161 ± 55^+^	934 ± 77*	750 ± 70*
IL-13	795 ± 81	1204 ± 89^+^	1155 ± 92	811 ± 98*
TNF-α	17 ± 11	115 ± 5^+^	100 ± 8	89 ± 14*

*Specific ELISA assays were employed for quantifications. Each value represents the mean ± SEM from seven to eight animals*.

*^+^P < 0.05 as compared to the saline-challenged group*.

*P < 0.05 as compared to the OVA-challenged group.

### Effects of 15d-PGJ_2_ on HDM-Induced Lung Inflammation and Remodeling

**Figure 5:**
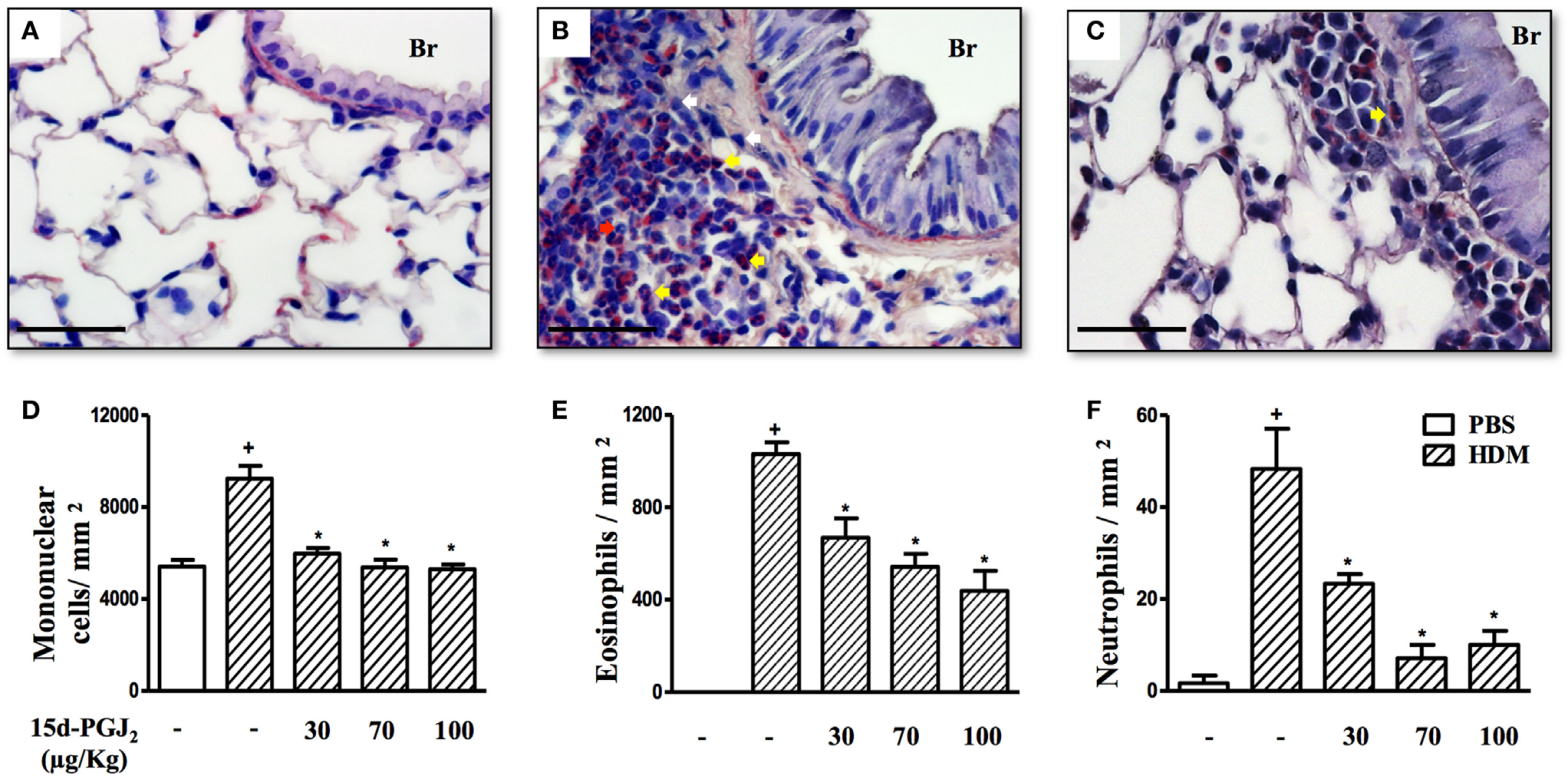
15d-PGJ_2_ decreases house dust mite extract (HDM)-induced peribronchiolar infiltration of leukocytes. Photomicrographs of representative airways in Sirius Red-stained lung sections from mice challenged with phosphate-buffered saline (PBS) **(A)**, HDM **(B)**, and HDM + 15d-PGJ_2_ (100 µg/kg, s.c.) **(C)**. White, yellow, and red arrows indicate mononuclear cell, eosinophil, and neutrophil, respectively. Peribronchiolar mononuclear cells **(D)**, eosinophils **(E)**, and neutrophils **(F)** were counted in 7–10 bronchioles per mouse. Treatments were carried out 30 min before HDM exposure during the last week of provocation. All samples for histologic examinations were undertaken 24 h after the last HDM challenge. Each value represents the mean ± SEM from six to seven animals. Br, bronchiolar lumen. Scale bar = 40 µm. ^+^*P* < 0.05 as compared to PBS-challenged group; *P < 0.05 as compared to HDM-challenged group.

**Figure 6:**
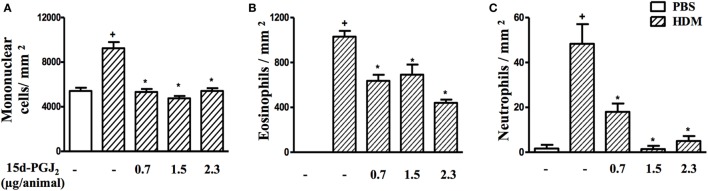
15d-PGJ_2_ given *via* nasal instillation reduces house dust mite extract (HDM)-induced peribronquiolar infiltration of leukocytes. Peribronchiolar mononuclear cells **(A)**, eosinophils **(B)**, and neutrophils **(C)** were counted in 7–10 bronchioles per mouse. 15d-PGJ_2_ (0.7–2.3 µg/mouse) was administered 30 min before HDM exposure during the last week of provocation. All samples for histologic examinations were undertaken 24 h after the last HDM challenge. Each value represents the mean ± SEM from five to seven animals. ^+^*P* < 0.05 as compared to phosphate-buffered saline (PBS)-challenged group; *P < 0.05 as compared to HDM-challenged group.

As expected, HDM provocation of A/J mice following the protocol described (Figure 1B) caused substantial peribronchiolar inflammatory infiltration as compared to sham-challenged mice (Figure 5B). Representative photomicrographs are shown in Figures 5A,B for negative and positive controls, respectively, whereas quantitative data are shown in Figures 5D–F. Differential analyses of these lung sections showed that leukocyte accumulation resulted from the recruitment of mononuclear cells (Figure 5D), eosinophils (Figure 5E), and neutrophils (Figure 5F). The interventional treatment of 15d-PGJ_2_ given *via* subcutaneous (30–100 µg/kg) (Figures 5C,D–F) or *via* intranasal instillation (0.7–2.3 µg/mouse) (Figures 6A–C) significantly inhibited the infiltration of mononuclear cells, eosinophils, and neutrophils as compared to vehicle treated mice.

**Figure 7:**
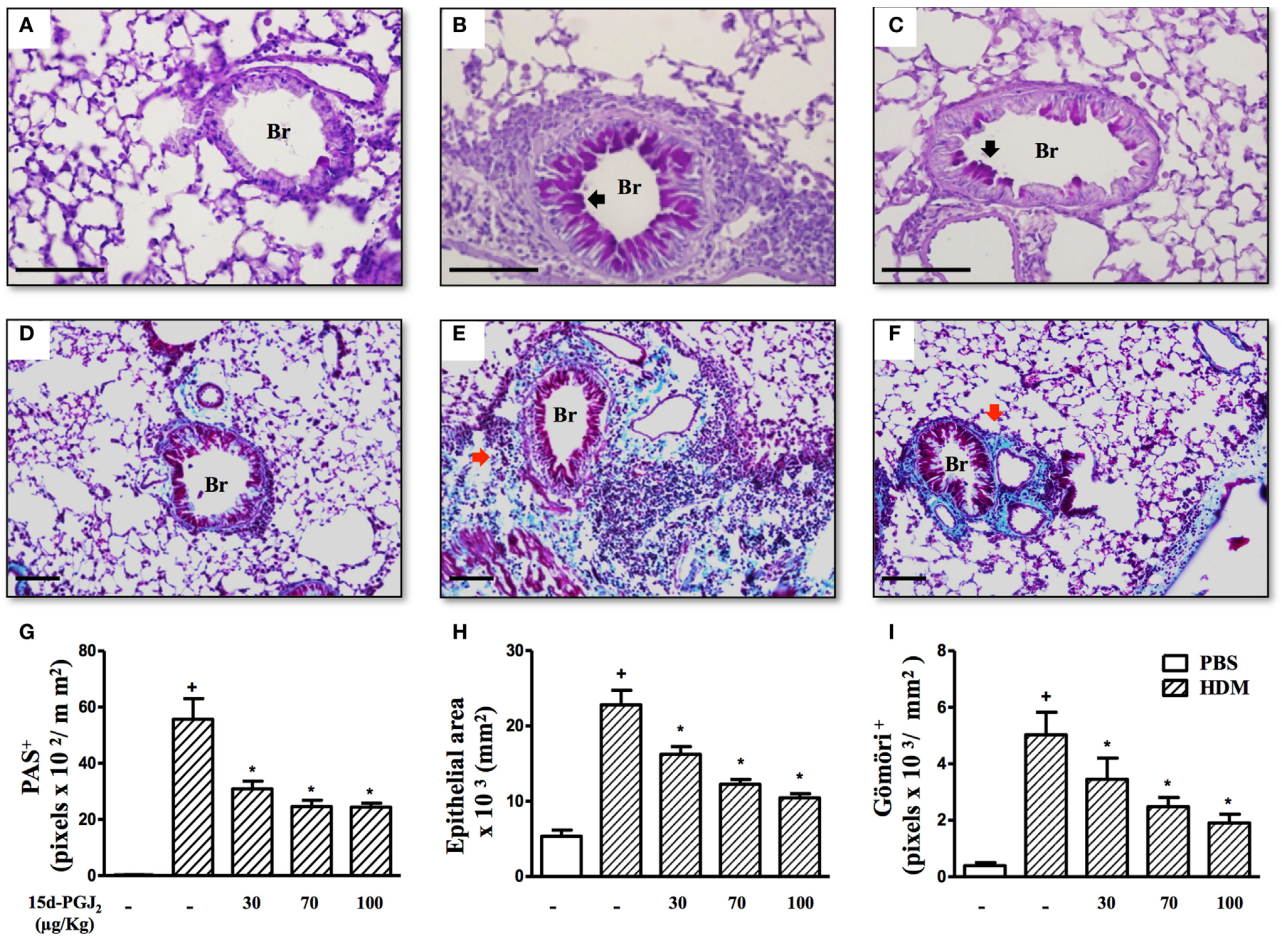
15d-PGJ_2_ inhibits house dust mite extract (HDM)-induced mucus exacerbation, bronchiolar epithelium thickening, and bronchiolar subepithelial fibrosis. Photomicrographs of representative airways in PAS-stained and Gömöri Trichrome-stained lung sections from mice challenged with phosphate-buffered saline (PBS) **(A,D)**, HDM **(B,E)**, and HDM + 15d-PGJ_2_ (100 µg/kg, s.c.) **(C,F)**. Black and red arrows indicate PAS and Gömöri Trichrome staining, respectively. Values of mucus production **(G)**, bronchiolar epithelium thickness **(H)**, and peribronchiolar extracellular matrix-elements deposition **(I)** were obtained by quantitative digital analyses in 8–10 airways per animal. Treatments were carried out 30 min before HDM exposure during the last week of provocation. All samples for histologic examinations were undertaken 24 h after the last HDM challenge. Each value represents the mean ± SEM from six to seven animals. Br, bronchiolar lumen. Scale bar = 100 µm. ^+^*P* < 0.05 as compared to PBS-challenged group; *P < 0.05 as compared to HDM-challenged group.

**Figure 8:**
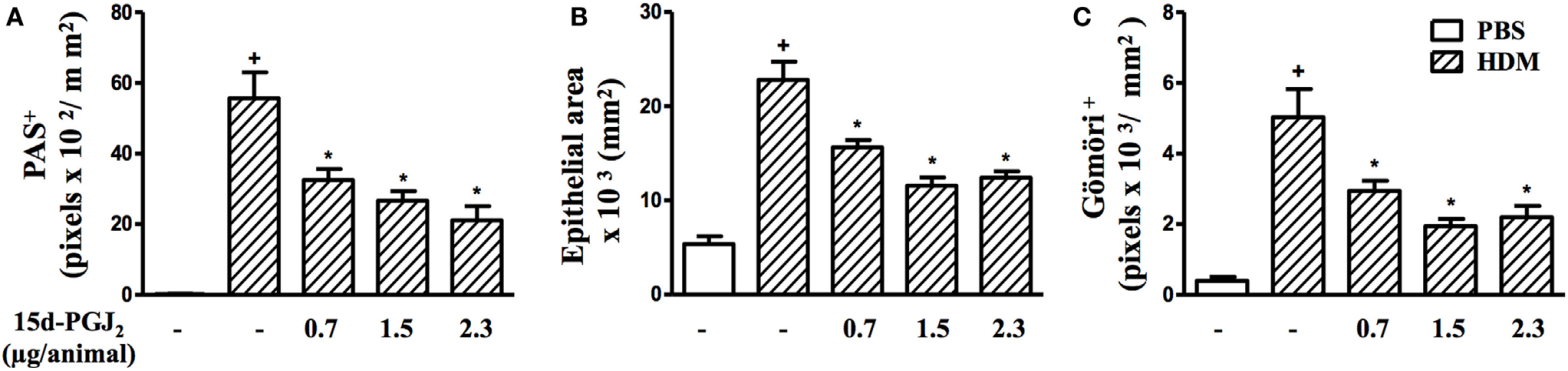
15d-PGJ_2_ given intranasally inhibits house dust mite extract (HDM)-induced mucus exacerbation, bronchiolar epithelium hypertrophy, and bronchiolar subepithelial fibrosis. Mucus production **(A)**, bronchiolar epithelium thickness **(B)**, and peribronchiolar fibrosis **(C)** were measured by quantitative digital analyses in 7–10 airways per mouse. 15d-PGJ_2_ (0.7–2.3 µg/mouse, i.n.) was administered 30 min before HDM exposure during the last week of provocation. All samples for histologic examinations were undertaken 24 h after the last HDM challenge. Each value represents the mean ± SEM from five to seven animals. ^+^*P* < 0.05 as compared to phosphate-buffered saline (PBS)-challenged group; *P < 0.05 as compared to HDM-challenged group.

Moreover, airways of HDM-challenged mice showed an increase in mucus production, epithelial area, and peribronchiolar fibrosis as compared to the PBS-challenged mice. Representative photomicrographs for mucus exacerbation and epithelial area are shown on Figures 7A,B, and for extracellular matrix deposition on Figures 7D,E as negative and positive controls are concerned, respectively. Quantitative values are shown in Figures 7G–I. Such changes were clearly sensitive to 15d-PGJ_2_ given *via* subcutaneous injection (30–100 µg/kg) (Figure 7C for mucus production and epithelial area, Figure 7F for peribronchiolar fibrosis, and Figures 7G–I, concerning quantitative data). Ongoing mucus exacerbation, epithelial thickening, and peribronchiolar fibrosis caused by HDM exposure were also inhibited by the local administration of 15d-PGJ_2_ (intranasal instillation, 0.7–2.3 µg/mouse) (Figures 8A–C).

### Effect of 15d-PGJ_2_ on HDM-Induced Airway Hyperreactivity

**Figure 9:**
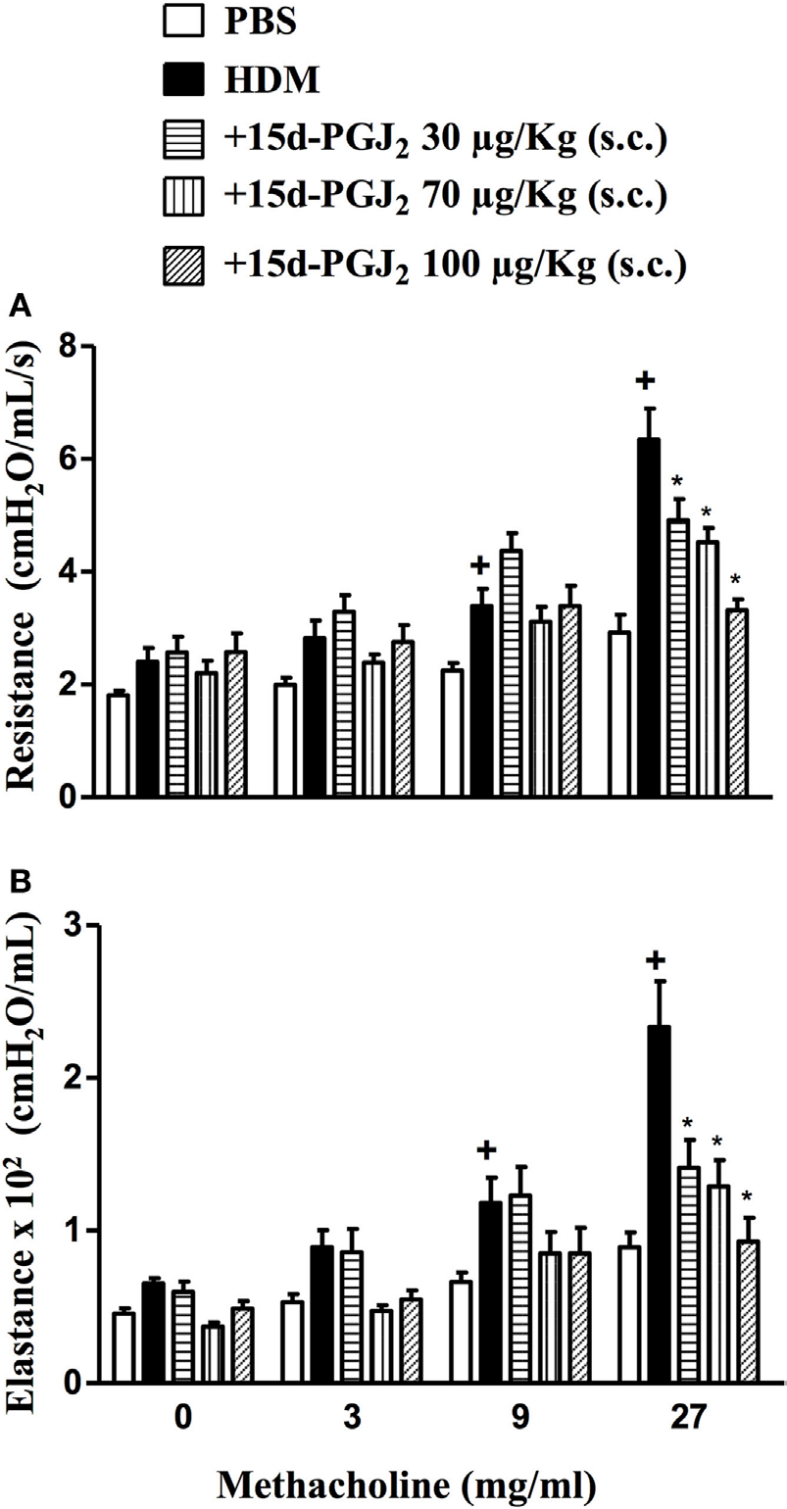
Subcutaneous administration of 15d-PGJ_2_ reduces house dust mite extract (HDM)-induced bronchial hyperreactivity. Airway responses were measured as changes in lung resistance **(A)** and elastance **(B)** induced by increasing concentrations of methacholine (3–27 mg/ml). Treatments were carried out 30 min before HDM exposure during the last week of provocation. The analyses were undertaken 24 h after the last HDM challenge. Each value represents the mean ± SEM from six to seven animals. ^+^*P* < 0.05 as compared to phosphate-buffered saline (PBS)-challenged group; *P < 0.05 as compared to HDM-challenged group.

Exposure to HDM resulted increased on levels of airway resistance and lung elastance following aerolized methacholine, as compared to PBS-challenged mice. Our findings revealed that 15d-PGJ_2_ at 30–100 µg/kg, s.c. dose-dependently decreased ongoing AHR concerning airway resistance (Figure 9A) and elastance (Figure 9B).

### Effects of 15d-PGJ_2_ on HDM-Induced Pro-inflammatory Cytokine and Chemokine Production

We evaluated the effect of the interventional treatment of 15d-PGJ_2_ (30–100 µg/kg, s.c.) on the HDM-induced elevation in the lung pro-inflammatory cytokines. As expected, mice exposed to HDM responded with significant increase in the levels of IL-5, IL-13, IL17, and eotaxin-1 compared to PBS-challenged mice, all of which being clearly abolished by 15d-PGJ_2_ (Table 2).

**Table 2:** 15d-PGJ_2_ (30–100 µg/kg, s.c.) decreases house dust mite extract (HDM)-induced cytokine and chemokine production in lung tissue samples.

Cytokine (ρg/lung)	Phosphate-buffered saline (PBS)	HDM	+15d-PGJ_2_ (30 µg/kg)	+15d-PGJ_2_ (70 µg/kg)	+15d-PGJ_2_ (100 µg/kg)
Interleukin (IL)-5	496 ± 68	787 ± 89^+^	480 ± 42*	435 ± 41*	386 ± 56*
IL-13	245 ± 35	413 ± 53^+^	250 ± 39*	192 ± 27*	228 ± 35*
IL-17	70 ± 6	107 ± 18^+^	57 ± 7*	55 ± 6*	51 ± 8*
Eotaxin-1	1,443 ± 107	12,210 ± 145^+^	4,174 ± 145*	6,411 ± 714*	5145 ± 558*

*Specific ELISA assays were employed for quantifications. Each value represents the mean ± SEM from four to seven animals*.

*^+^P < 0.05 as compared to the PBS-challenged group*.

*P < 0.05 as compared to the HDM-challenged group.

### Effect of 15d-PGJ_2_ on HDM-Induced NF-κB Expression and Activation in the Lung

**Figure 10:**
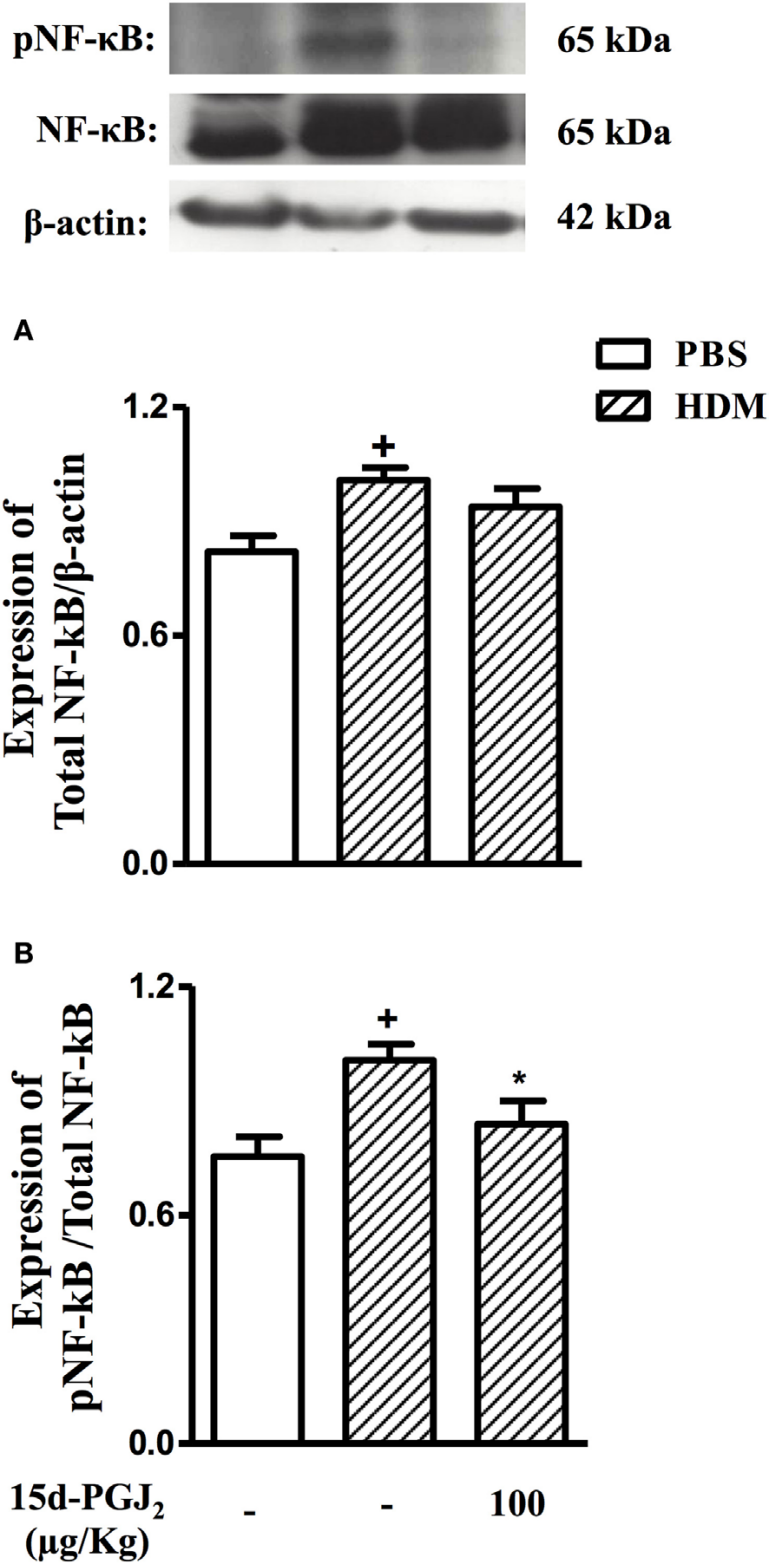
15d-PGJ_2_ (100 μg/kg, s.c.) reduces phosphorylation of NF-κB in mice exposed to house dust mite extract (HDM). Pulmonary expression of NF-κB **(A)** and pNF-κB **(B)** were assessed by western blotting and quantified by densitometry. NF-κB expression was normalized to β-actin while pNF-κB expression was normalized to total NF-κB. Each blot is representative of three identical experiments. Each value represents the mean ± SEM from four animals. ^+^*P* < 0.05 as compared to phosphate-buffered saline (PBS)-challenged group; *P < 0.05 as compared to HDM-challenged group.

As compared to sham-challenged mice, those exposed to HDM responded with increased expression and activation of NF-κB in the lung (Figure 10). 15d-PGJ_2_ (100 µg/kg, s.c.) inhibited NF-κB phosphorylation (Figure 10A) without modifying NF-κB expression (Figure 10B) triggered by allergen provocation.

### Lack of Effect of GW9662 on Mast Cell Stabilizing Properties Promoted by 15d-PGJ_2_

**Figure 11:**
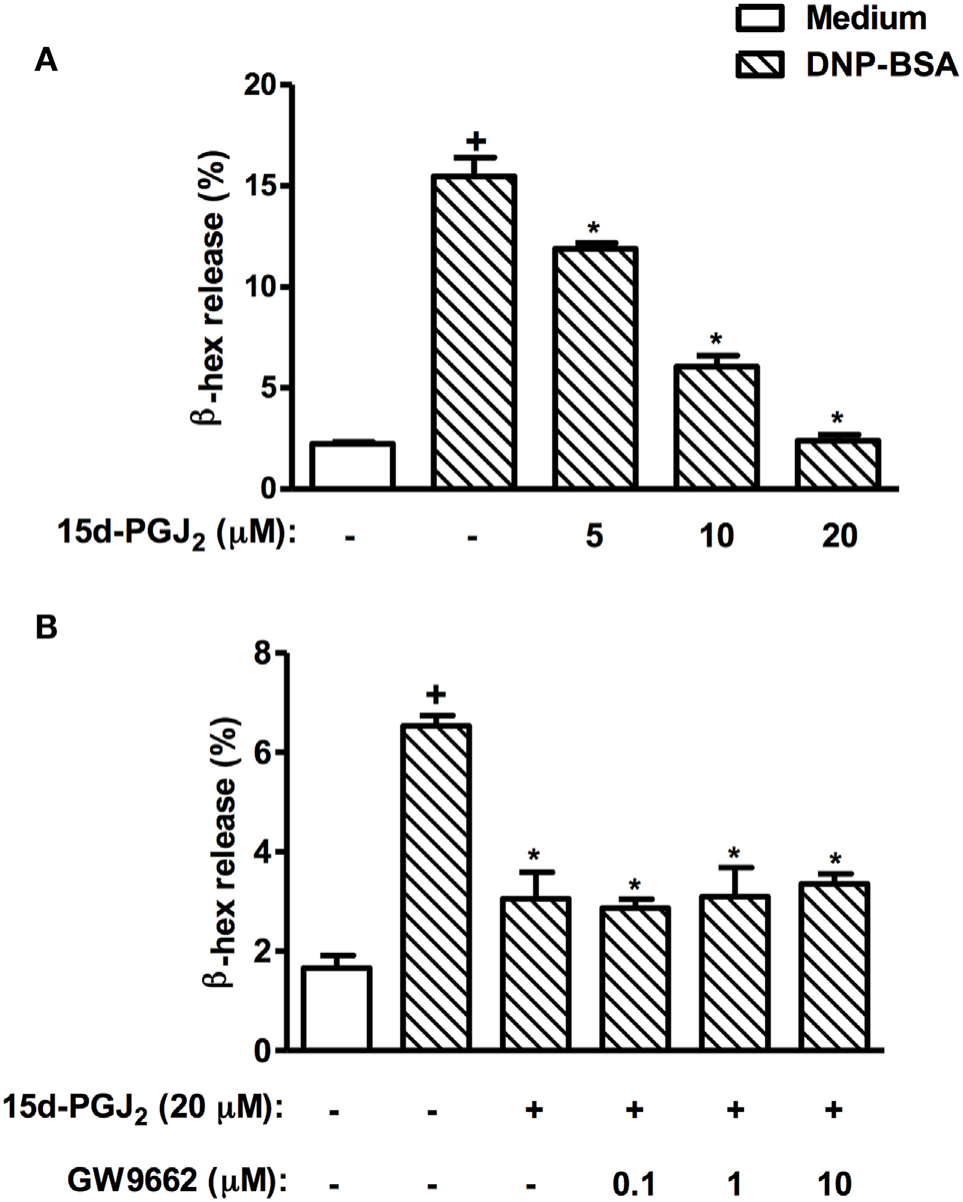
15d-PGJ_2_ inhibits mast cell degranulation *in vitro*. RBL-2H3 cells (1.25 × 10^5^ cells/well) were sensitized with IgE anti-DNP (1 µg/ml), incubated for 20 h, and treated with 15d-PGJ_2_ (5–20 µM) **(A)**, pretreated or not with GW662 (0.1–10 µM) **(B)**, and stimulated with DNP–BSA (10 ng/ml) 45 min later. The β-hexosaminidase levels were quantified 45 min after stimulus using a spectrophotometer at 405 nm. These results are expressed as the mean ± SEM from four independents experiments. ^+^*P* < 0.05 as compared to unchallenged group; *P < 0.05 as compared to DNP–BSA challenged group.

In an *in vitro* setting, we explored the effect of 15d-PGJ_2_ on allergen-induced mast cell degranulation as a way of assessing the potential role of PPAR-γ on the 15d-PGJ_2_’s anti-allergic action. As shown in Figure 11A, 15d-PGJ_2_ (5–20 µM) dose-dependently inhibited DNP-BSA-induced mast cell degranulation, in a mechanism clearly resistant to the PPAR-γ antagonist GW9662 (10 µM) (Figure 11B).

## Discussion

In this study, we have done investigations into the putative capacity of the PPAR-γ ligand 15d-PGJ_2_ to reverse ongoing lung pathological changes triggered by allergen in two distinct mouse models of asthma. We demonstrated for the first time that the interventional treatment with this 15d-PGJ_2_, given either systemically or locally, inhibits ongoing lung pathological features triggered by either OVA or HDM extract. The treatment inhibited allergen-induced eosinophilic and neutrophilic inflammatory infiltrate, epithelial thickening, mucus overproduction, and peribronchiolar fibrosis. We showed a correlation between anti-inflammatory and anti-remodeling effects of 15d-PGJ_2_ with reduction in the levels of crucial pro-inflammatory cytokines. Furthermore, treatment with 15d-PGJ_2_ inhibited HDM-induced AHR and activation of NF-κB in the lung, suggesting that this compound does hold promising perspectives as alternative in drug development for asthma.

There are several clinical clues advancing PPAR-γ as a relevant target in asthma therapy (41, 42). In addition, prior studies revealed that synthetic PPAR-γ agonists, including rosiglitazone, pioglitazone, and ciglitazone, have beneficial effects on airway inflammation in distinct animal models of asthma, due to the pleiotropic role of the PPAR-γ pathway activation (43, 44). However, the use of potent synthetic PPAR-γ agonists in disease management requires precaution, since activation of PPARs alters the transcription of a multitude of genes in various organs, raising about the possibility of a variety of adverse effects as consequence (19, 20). Remarkably, rosiglitazone, and other PPAR-γ ligands, were withdrawn from the European market because of adverse side effects (19, 20). On the other hand, it is noteworthy that the endogenous prostaglandin 15d-PGJ_2_ mediates many of the anti-inflammatory actions associated with activation of PPAR-γ (45, 46) and might be a safer therapy for asthma patients as compared with synthetic PPAR-γ agonists.

Mouse models of inflammatory airway disease based on the allergenic potential of HDM extract or OVA have been important for the assessment of asthma targets and treatments in ways that would not be feasible in humans. The advantage of HDM over OVA is that pathological abnormalities result from mucosal sensitization within the lungs, as occurring in humans, and does not require systemic adjuvant sensitization (2). In our study, therapeutic treatment with 15d-PGJ_2_ (30–100 µg/kg, s.c.) initiated 2 weeks after HDM nasal instillations were equally active in inhibiting the peribronchiolar infiltration of inflammatory cells, such as eosinophils, neutrophils, and mononuclear cells. Similarly, it was effective in reversing peribronchiolar accumulation of eosinophils, neutrophils, and AHR in an OVA-induced murine model of asthma. Indeed, eosinophils have been described as crucial effector cells in asthmatic lung damage and AHR (47). Neutrophils are also present in more severe cases of asthma, contributing to tissue damage and subsequent perpetuation of asthmatic framework (47). The protective effect of 15d-PGJ_2_ on neutrophil infiltration is consistent with a prior work, in which administration of this compound limited the influx of neutrophils in airways of mice with lung injury induced by bleomycin (48). There has long been controversy whether or not lung inflammation correlates, in a causal manner, with airway hyperreactivity in asthmatic patients (49). In the current study, 15d-PGJ_2_ (30–100 µg/kg, s.c.) inhibited significantly both HDM-induced leukocyte recruitment and AHR, suggesting that these events might be somehow correlated at least in this animal model.

Thickening of the airway epithelium layer due to hypertrophy and hyperplasia of mucin-secreting goblet cells, as well as collagen deposition, are probably the most significant features of airway remodeling, particularly in severe asthmatic patients (49, 50). Our findings revealed that 15d-PGJ_2_ dose-dependently reversed structural changes, such as epithelial thickening, mucus exacerbation, and extracellular matrix deposition in mice exposed to HDM or OVA. These findings are supported by studies in human lung fibroblasts, which demonstrated the effect of 15d-PGJ_2_ in attenuation of proliferation and differentiation of fibroblasts into myofibroblasts. This is relevant since myofibroblasts are sources of extracellular matrix proteins, primarily collagen, as well as cytokines and chemokines, related to subepithelial fibrosis (51). Moreover, *in vitro* studies have demonstrated that 15d-PGJ_2_ can inhibit human cultured airway smooth muscle cell proliferation in response to basic fibroblast growth factor (52).

Interleukin (IL)-13 is a Th2 cytokine associated with AHR, IgE production, and airway remodeling (53). In our hands, in both OVA and HDM mouse models, exogenous 15d-PGJ_2_ inhibited allergen-induced levels of IL-13 in parallel with significant decrease of mucus production and peribronchiolar fibrosis, reinforcing the interpretation that inflammation is underlying the lung remodeling in these systems. In our conditions, irrespective of the mouse model utilized, 15d-PGJ_2_ therapeutic treatment significantly reduced the lung tissue levels of eotaxin-1 and IL-5 in parallel with reduction in eosinophil counts, which is in line with the fact that these mediators strongly stimulate production, chemotaxis, and activation of eosinophils (54). IL-17 is a Th17 cytokine, often present in severe asthma patients, involved in the development of airway neutrophilia (55). Our results indicate that attenuation of neutrophil influx by subcutaneous 15d-PGJ_2_ was accompanied by reduction of lung tissue levels of IL-17 in animals challenged with HDM suggesting a positive correlation between these events. In addition, the treatment with 15d-PGJ_2_ reduced the levels of TNF-α, a pro-inflammatory cytokine known to promote accumulation and activation of granulocytes, and also able to underline fibrotic processes *via* induction of myofibroblast proliferation (56). This result is consistent with prior investigations showing that TNF-α production by murine macrophages *in vitro* is also sensitive to 15d-PGJ_2_ (57).

It should be emphasized that the effect of systemic treatment with 15d-PGJ_2_ could be also replicated following topical administration under conditions of HDM-induced asthma changes. The advantage of a topical treatment is that the drug is delivered directly to the target organ, thereby bypassing pharmacokinetic factors, which can significantly influence the tissue concentrations (58). In fact, intranasal instillation of 15d-PGJ_2_ was as effective as the subcutaneous administration in reducing both allergic inflammation and airway remodeling. One plausible explanation is that 15d-PGJ_2_ is catabolized mainly through conjugation with glutathione in the liver (59). The routes of administration used in this study, unlike the oral treatment, do not undergo first-pass metabolism, thus, there is a relative lower exposure of 15d-PGJ_2_ to the liver glutathione, preserving the quantity of the drug administered.

Although PPAR-γ is thought to be the primary target of 15d-PGJ_2_ in various of its actions, there are also convincing evidence that this prostanoid can act independently of PPAR-γ. Since mast cells are pivotal elements in triggering allergen-induced inflammatory changes (60), we used a classic system of IgE-sensitized RBL-2H3 cells to study the potential implication of PPAR-γ on the anti-allergic effect presented by 15d-PGJ_2_. Our findings revealed that 15d-PGJ_2_ was able to inhibit dose-dependently allergen-induced mast cell degranulation in a mechanism clearly unaffected by the PPAR-γ antagonist GW9662. There are indeed a robust body of evidence in the literature indicating that 15d-PGJ_2_ can promote *in vitro* apoptosis of eosinophils (28) and downregulation of IL-13 production by T cells (29) *via* PPAR-γ-independent mechanisms. In both cases, interference with NF-κB nuclear translocation seems to underlie the effects. Accordingly, we demonstrated in this study that the therapeutic treatment with 15d-PGJ_2_ clearly attenuated the expression of phosphorylated NF-κB in the lung extract obtained from mice subjected to chronic exposure of HDM. Since NF-κB pathway is highly expressed in severe asthmatics (27), and it accounts for the upregulation of pivotal pro-inflammatory cytokines in asthma pathogenesis, such as IL-13 and TNF-α (29), it is not unlikely that the anti-asthmatic effect of 15d-PGJ_2_ is mediated, at least in part, *via* a NF-κB-dependent mechanism.

The experiments reported here demonstrate that exogenous 15d-PGJ_2_, given either systemically or locally, can control ongoing asthma pathological abnormalities, including eosinophil and neutrophil infiltration, AHR, mucus exacerbation, and lung remodeling triggered by either ovalbumin or HDM. Inhibition of the NF-κB signaling pathway and subsequent downregulation of pivotal pro-inflammatory cytokines seems to be implicated in this mechanism. Our findings suggest that potential exists to exploit 15d-PGJ_2_ as therapeutic agent in asthma. However, new experiments are required in order to assess crucial parameters of toxicity and safety following systemic and topical administration of this compound.

## Ethics Statement

The Animal Ethics Committee of the Oswaldo Cruz Foundation approved all procedures involving care and use of animals in this study (license no LW 23/10).

## Author Contributions

DC: acquisition and analysis of data, illustration, revision for intellectual content, and final approval. EA-V: acquisition and analysis of data, revision for intellectual content, and final approval. CN: acquisition and analysis of data and revision for intellectual content and final approval. AP: acquisition and analysis of data, revision for important intellectual content, illustration, and final approval. MN: contributions to design of the work, revision for intellectual content, and final approval. VC: acquisition and analysis of data, providing illustration, revision for important intellectual content, and final approval. RT: acquisition and analysis of data and final approval. PS: contributions to design of the work, illustration, critical revision, supervision, and final approval. MM: design of the study, revision for intellectual content, illustrations, and final approval.

## Conflict of Interest Statement

The authors declare that the research was conducted in the absence of any commercial or financial relationships that could be construed as a potential conflict of interest.
